# Commentary: What have we learnt about the sourcing of personal protective equipment during pandemics? Leadership and management in healthcare supply chain management: a scoping review

**DOI:** 10.3389/fpubh.2025.1701145

**Published:** 2026-01-12

**Authors:** T. Balamurugan, M. N. Mohamed Sadiq, Ancy Antony, Meghana Rajan

**Affiliations:** 1 SRM Institute of Science and Technology - Vadapalani Campus, Chennai, India; 2 Joy University, Tirunelveli, India; 3 PSG College of Arts and Science, Coimbatore, India

**Keywords:** COVID-19, leadership, pandemic, personal protective equipment (PPE), resilience, supply chain, supply chain management (SCM)

## 1 Introduction

Best and Williams (1) conducted a timely scoping review that examined the sourcing of Personal Protective Equipment (PPE) during pandemics, particularly COVID-19. Their review illuminated systemic fragilities in healthcare supply chains, including over-reliance on single suppliers, lack of stockpiles, and limited resilience. By synthesizing lessons across earlier outbreaks, they underscored the importance of leadership, planning, and collaboration in shaping preparedness. This commentary builds on their contribution by extending the discussion into three crucial domains: (1) digital innovations for resilient supply chains, (2) sustainability and the environmental dimensions of PPE sourcing, and (3) preparedness through simulation modeling and Industry 4.0 approaches. These perspectives highlight that future strategies must move beyond reactive procurement and stockpiling to embrace integrated, technology-driven, and environmentally conscious systems.

### 1.1 Sourcing challenges and contributions of the review

The scoping review convincingly documented disruptions such as scarcity, inflation in prices, and inequities in distribution (1). The authors noted how just-in-time models faltered when sudden demand spikes emerged, and they emphasized the need for diversified suppliers and stronger governance. These findings align with broader literature on supply chain vulnerabilities during COVID-19, which identified transparency deficits, stockpiling inefficiencies, and inadequate international coordination (2, 3). By situating PPE challenges within leadership and management contexts, the review provides a governance perspective that moves beyond technical supply chain analysis and highlights relational and decision-making factors.

### 1.2 Digital innovations in PPE supply chains

While the review was published in 2021, subsequent research has advanced our understanding of digital transformation in supply chains. Artificial intelligence (AI) platforms now provide predictive analytics, enabling real-time visibility of PPE demand and distribution (15). Blockchain technologies further enhance transparency by securing traceability across suppliers, reducing the risk of counterfeit or substandard equipment (4). Recent evidence suggests that organizations with robust digital infrastructures experienced fewer disruptions and greater agility during the COVID-19 pandemic (5). These tools are no longer optional add-ons but essential enablers for resilient healthcare supply chains. Integrating digital “control towers” can allow policymakers and healthcare leaders to anticipate shortages and dynamically reconfigure sourcing strategies. Beyond conceptual advantages, empirical evidence suggests that hospitals implementing AI-driven demand forecasting during the COVID-19 pandemic experienced significant reductions in PPE stock-outs, while blockchain-enabled traceability systems helped curb the circulation of counterfeit PPE within procurement networks (6). These findings substantiate the operational necessity of digital tools for resilient healthcare supply chains rather than their use as purely strategic enhancements. At the same time, practical challenges constrain large-scale adoption, particularly in low- and middle-income settings. High implementation costs, limited digital infrastructure, lack of technical expertise, weak system interoperability, and fragmented regulatory frameworks continue to restrict the effective deployment of advanced digital supply chain technologies across healthcare systems.

### 1.3 Sustainability and environmental dimensions

A second critical lens is sustainability, which was not a major focus of the scoping review but has since become unavoidable. The surge in single-use PPE has generated significant environmental costs, with plastic-based debris now recognized as a pollutant in multiple ecosystems (7). Studies using life cycle assessment approaches emphasize the urgent need to shift toward reusable or recyclable PPE, incorporating antimicrobial coatings and durable materials (8, 9). The circular economy model provides a promising framework by keeping resources in use, reducing waste, and fostering closed-loop supply systems. For future pandemics, resilience cannot be divorced from sustainability; the legitimacy of public health responses increasingly depends on balancing safety with environmental responsibility. However, sustainable PPE solutions remain unevenly accessible across regions. Resource-constrained health systems frequently depend on low-cost disposable PPE and have limited waste-management capacity, exacerbating environmental and occupational health risks. This asymmetry reinforces global inequities in both environmental exposure and workforce protection (14).

### 1.4 Preparedness through simulation and Industry 4.0

Preparedness strategies must also expand beyond stockpiling toward predictive modeling and Industry 4.0 solutions. Agent-based simulations that integrate epidemiological dynamics with supply logistics can stress-test vulnerabilities and guide trade-offs between short-term costs and long-term robustness (10). Meanwhile, Industry 4.0 technologies such as additive manufacturing and 3D printing demonstrated their potential during COVID-19 by filling critical PPE gaps. Although scalability and regulatory compliance remain challenges, localized production can buffer global supply shocks. These approaches shift preparedness from reactive contingency planning to proactive scenario-building, offering decision-makers greater agility in responding to uncertain future crises. Notwithstanding these benefits, decentralized production models face regulatory, quality assurance, and standardization challenges that may limit their immediate clinical scalability. The absence of harmonized international certification pathways for emergency-manufactured PPE continues to constrain rapid deployment during crises.

### 1.5 Equity, access, and policy barriers in PPE supply chains

The COVID-19 pandemic revealed profound inequities in global access to PPE, with frontline healthcare workers in low-income regions experiencing chronic shortages while high-income countries secured large stockpiles through advance purchasing agreements and export controls (11). These asymmetries exposed structural power imbalances within global PPE markets and undermined equitable health protection. Policy failures further intensified access disparities. Weak global coordination, inconsistent national emergency procurement regulations, and the absence of binding international allocation mechanisms impeded fair distribution during peak crisis periods. Establishing regional manufacturing hubs, multilateral stockpile agreements, and transparent global allocation frameworks may mitigate these inequities during future emergencies (12). Financial constraints remain a central barrier to equitable preparedness. Many health systems lack the fiscal capacity to invest in digital infrastructure, sustainable PPE alternatives, and strategic reserves. International financing mechanisms, technology-transfer partnerships, and public–private collaborations are therefore essential for closing persistent preparedness gaps (13).

## 2 Discussion

Taken together, the insights from Best and Williams (1) and subsequent scholarship reveal a multidimensional picture of PPE supply chain resilience. The scoping review's emphasis on leadership, planning, and collaboration remain central. However, resilience must now be reframed to incorporate digital transformation, sustainability, and predictive preparedness. These elements are mutually reinforcing: digital visibility enhances coordination, sustainable design mitigates waste and dependence on single-use items, and simulations allow leaders to anticipate disruptions before they escalate. The integration of these approaches represents a paradigm shift from fragmented procurement to holistic system resilience. Nevertheless, counterarguments warrant serious consideration. Over-reliance on algorithmic forecasting systems may introduce systemic vulnerabilities if models fail under unprecedented demand patterns. Blockchain infrastructures raise concerns regarding scalability, data governance, cybersecurity, and energy consumption. Similarly, sustainable PPE alternatives often involve higher upfront costs and uncertainties regarding long-term durability in intensive clinical settings. These trade-offs emphasize the need for context-specific, balanced adoption strategies rather than universal technological prescriptions.

## 3 Conclusion

Best and Williams (1) provided a crucial baseline by mapping persistent fragilities in PPE supply chains and emphasizing the role of leadership in overcoming them. Building on their work, this commentary argues that future resilience strategies must extend beyond traditional stockpiling and diversification. Policymakers and healthcare leaders should embrace AI-enabled visibility tools, blockchain-based traceability, and predictive simulations, while also embedding sustainability and circular economy principles. By aligning leadership, technology, and environmental stewardship, healthcare systems can transition from reactive crisis management to proactive, adaptive, and sustainable preparedness. Such a transformation is essential not only for navigating the next pandemic but also for ensuring long-term resilience in global health supply chains. Future studies should focus on empirically testing the effectiveness of digital supply chain tools such as AI-driven forecasting and blockchain-enabled transparency in healthcare contexts. Research is also needed on developing and scaling sustainable PPE solutions, particularly reusable and recyclable alternatives, within circular economy frameworks. Additionally, simulation-based modeling and localized manufacturing (e.g., 3D printing) should be further examined to evaluate their scalability, cost-efficiency, and regulatory feasibility. Exploring these areas will not only extend the contribution of Best and Williams (1) but also provide practical guidance for policymakers and organizations preparing for future global health crises. Realizing these goals will require overcoming substantial financial, infrastructural, and policy barriers. Without sustained public investment, regulatory harmonization, and inclusive global governance mechanisms, the benefits of digitalization and sustainable PPE innovations risk remaining unevenly distributed across health systems.
